# COVID-19: Lung-Centric Immunothrombosis

**DOI:** 10.3389/fcimb.2021.679878

**Published:** 2021-06-11

**Authors:** Peter R. Kvietys, Hana. M. A. Fakhoury, Sana Kadan, Ahmed Yaqinuddin, Eid Al-Mutairy, Khaled Al-Kattan

**Affiliations:** ^1^ College of Medicine, Alfaisal University, Riyadh, Saudi Arabia; ^2^ Department of Medicine, King Faisal Specialist Hospital and Research Centre (KFSHRC), Riyadh, Saudi Arabia

**Keywords:** acute respiratory distress syndrome (ARDS), coronavirus, COVID-19, cytokine storm, NET, SARS-CoV-2

## Abstract

The respiratory tract is the major site of infection by SARS-CoV-2, the virus causing COVID-19. The pulmonary infection can lead to acute respiratory distress syndrome (ARDS) and ultimately, death. An excessive innate immune response plays a major role in the development of ARDS in COVID-19 patients. In this scenario, activation of lung epithelia and resident macrophages by the virus results in local cytokine production and recruitment of neutrophils. Activated neutrophils extrude a web of DNA-based cytoplasmic material containing antimicrobials referred to as neutrophil extracellular traps (NETs). While NETs are a defensive strategy against invading microbes, they can also serve as a nidus for accumulation of activated platelets and coagulation factors, forming thrombi. This immunothrombosis can result in occlusion of blood vessels leading to ischemic damage. Herein we address evidence in favor of a lung-centric immunothrombosis and suggest a lung-centric therapeutic approach to the ARDS of COVID-19.

## Introduction

SARS-CoV-2, the coronavirus responsible for COVID-19, initially infects the nasal, bronchial and alveolar epithelial cells (Hoffmann et al., 2020; Milewska et al., 2020; Zhu et al., 2020). Resident immune cells such as macrophages and dendritic cells are also infected, albeit to a lesser extent (Carsana et al., 2020; Schaefer et al., 2020; Yang et al., 2020). The SARS-CoV-2 pulmonary tropism is manifested in the clinical features of COVID-19 (e.g., cough, dyspnea). The clinical course of SARS-CoV-2 infection is subdivided into three outcomes. Most patients will be asymptomatic or display minor respiratory symptoms and recover without hospitalization. Roughly 10-20% of affected individuals will advance to pneumonia/hypoxia and require hospitalization, but will eventually recover (Chowdhury et al., 2021; Morris et al., 2021). Only a small fraction (5 - 10%) of COVID-19 patients will progress to acute respiratory distress syndrome (ARDS) and require aggressive treatment in intensive care units (e.g., mechanical ventilation) (Chowdhury et al., 2021; Morris et al., 2021). Some of these patients will eventually succumb to the disease with the major cause of death being respiratory failure (Ackermann et al., 2020; Phua et al., 2020; Tay et al., 2020). Autopsy findings indicate that the lungs bear the greatest pathologic burden, characterized by diffuse alveolar damage, inflammatory cell infiltrates and thrombosis (Ackermann et al., 2020; Lax et al., 2020; Liu et al., 2020; Deshmukh et al., 2021). Of note, organs remote from the initial site of infection such as the heart and kidneys are not spared. Histopathologic features in autopsy specimens of these remote organs include the presence of microvascular thrombi adjacent to regions of necrosis (Ackermann et al., 2020; Rapkiewicz et al., 2020; Tang et al., 2020). Thus, in severe COVID-19 there is evidence indicative of an hypercoagulative state and multiorgan dysfunction.

A dysregulated immune response to SARS-CoV-2 infection is believed to play a major role in the pathogenesis of COVID-19. Specifically, there is an impaired antiviral response in conjunction with an excessive inflammatory response (Blanco-Melo et al., 2020; Hadjadj et al., 2020). Lymphopenia is common and may be coupled to markers of T cell exhaustion in the circulation and decreased numbers in lymphoid tissues (Diao et al., 2020; Giamarellos-Bourboulis et al., 2020; Lee et al., 2020; Liu et al., 2020; Lucas et al., 2020; Zhao et al., 2020; Zheng et al., 2020; Ronit et al., 2021). An inadequate lymphocyte-mediated antiviral response would protract the viral infection and thereby exacerbate the inflammatory response (Lee et al., 2020; Lucas et al., 2020; Manjili et al., 2020; Ronit et al., 2021).

## Lung-Centric Inflammation

Analyses of bronchoalveolar lavage fluid (BALF) of COVID-19 patients indicate a pro-inflammatory environment within the lungs (De Biasi et al., 2020; Liao et al., 2020; Pandolfi et al., 2020; Wang et al., 2020a; Ronit et al., 2021). Their BALF is enriched in pro-inflammatory chemokines and cytokines as well as activated macrophages and neutrophils. Chemokines (e.g., IL-8) dominate the BALF profile in moderate cases of COVID-19, whereas cytokines (e.g., TNF-α, IL-6) are prevalent in more severe cases (Liao et al., 2020).

**Figure 1:**
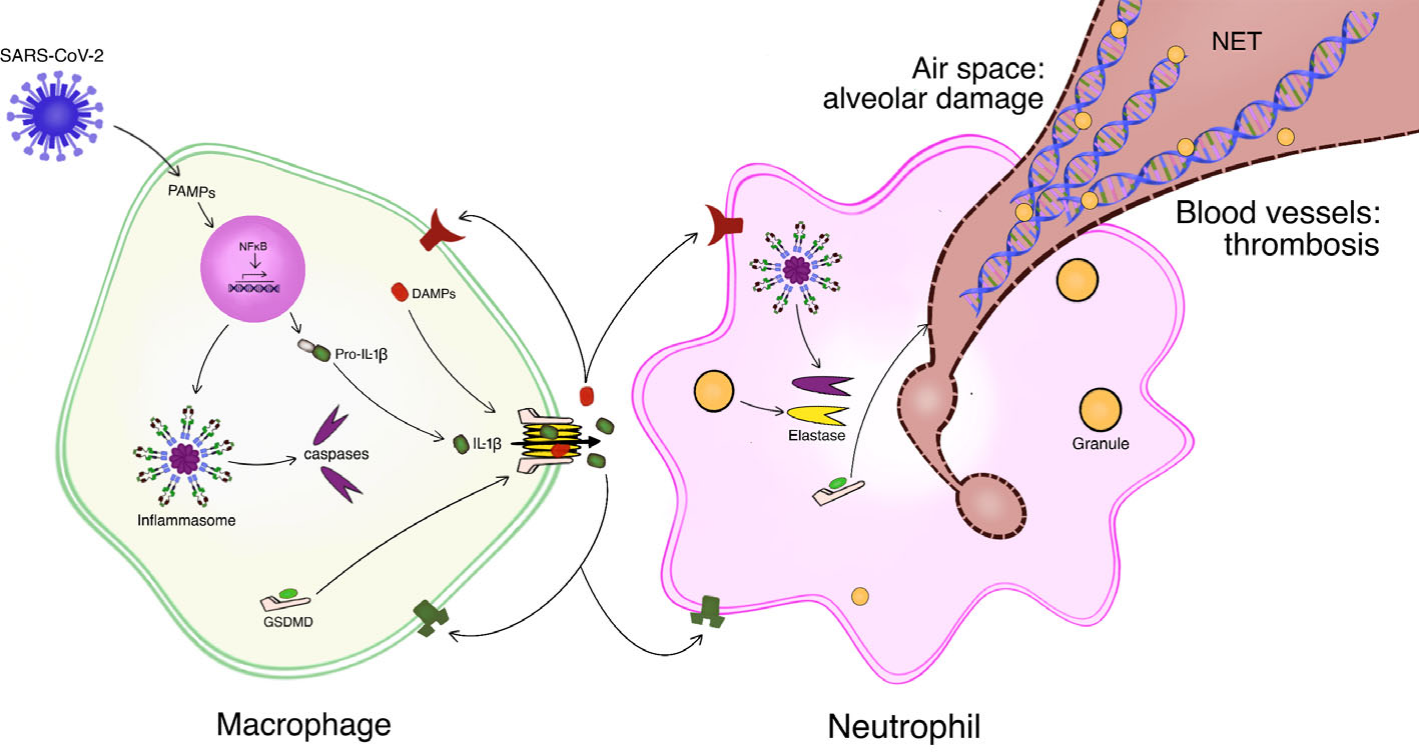
Schematic of lung-centric inflammation and immunothrombosis in response to SARS-CoV-2 infection. Resident alveolar macrophages mount an inflammatory response to infection of the lungs by SARS-CoV-2. Macrophages detect viral material e.g., pathogen-associated molecular patterns (PAMPs). PAMPs activate the NFκB pathway which generates pro-IL-1β and components of the inflammasome. Assembly and functional activation of the inflammasome results in caspase-mediated cleavage of pro-IL-1β to the mature IL-1β. Caspase cleavage of gasdermin D (GSDMD) allows it to enter the plasma membrane and oligomerize, forming pores. IL-1β as well as other inflammatory mediators, such as damage-associated molecular patterns (DAMPs) exit the macrophage through the GSDMD pores. The inflammatory response is amplified by feed-forward mechanisms and recruitment of additional leukocytes, e.g., neutrophils. Activated neutrophils can extrude neutrophil extracellular traps (NETs), a meshwork of decondensed DNA decorated with granule-derived proteases and antimicrobials. Inflammasome-derived caspase as well as granule-derived elastase activate GSDMD to form the pores for NET release. NETs formed in the alveolar space can induce lung injury while NETs generated within blood vessels sequester platelets and coagulation factors to promote thrombogenesis. Modified from (Tall and Westerterp, 2019).

A major cytokine involved in initiation and progression of the inflammatory response is IL-1β, the activation of which is dependent on the NLRP3 inflammasome (Kelley et al., 2019; Swanson et al., 2019; Zhao and Zhao, 2020). In brief, detection of viral material (e.g., PAMPs) by alveolar macrophages activates the NFκB transcription pathway, resulting in the generation of nascent pro-IL-1β as well as components of the NLRP3 inflammasome (**Figure 1**). Subsequently, the inflammasome is assembled and serves as a platform for caspase-mediated cleavage of pro-IL-1β to the mature form. IL-1β lacks a signal sequence and is retained in the cytoplasm until an exit portal is created in the plasma membrane. To this end, gasdermins, also activated by the caspases, enter the plasma membrane and oligomerize to form pores (Broz et al., 2020). IL-1β and other pro-inflammatory material (e.g., DAMPs) exit *via* these gasdermin pores. Collectively, the released mediators amplify the local inflammatory response *via* feed-forward mechanisms, including cytokine-induced cytokine release and recruitment of additional innate immune cells (e.g., neutrophils).

The NFκB pathway and the NLRP3 inflammasome appear to be operative in COVID-19 patients (Lee et al., 2020; Hariharan et al., 2021) and may contribute to lethality (Lara et al., 2020). SARS-CoV-2 infects human monocytes and activates the NLRP3 inflammasome, resulting in gasdermin-mediated pyroptosis (Ferreira et al., 2021). Active caspases and cytokines are present in the circulation of these patients, with higher levels noted in severe cases (Rodrigues et al., 2021). Further, inflammasome components are detected in autopsy specimens of lung tissue, primarily in macrophages and to a lesser extent in alveolar epithelia (Rodrigues et al., 2021; Toldo et al., 2021).

## Lung-Centric “Cytokine Storm”

The cytokines and chemokines generated within the lungs in response to infection can spill into the general circulation, a convenient sampling reservoir for both clinical and experimental purposes. Based on elevated circulating levels of cytokines, the term “cytokine storm” has been used as a descriptor of the hyperinflammatory response involved in the pathogenesis of COVID-19 (Barnes et al., 2020; Castelli et al., 2020; Fajgenbaum and June, 2020; Mehta et al., 2020; Merad and Martin, 2020; Tay et al., 2020). This descriptor has been challenged on the grounds that the measured blood levels of inflammatory cytokines in COVID-19 patients are orders of magnitude less than levels reported in other cases of ARDS such as sepsis or influenza (Kox et al., 2020; Mudd et al., 2020; Sinha et al., 2020). The issue is further confounded by a lack of a precise definition for the term “cytokine storm” (Fajgenbaum and June, 2020). A reasonable approach to avoid this rather semantic issue is to consider the local pulmonary storm as being more severe than the systemic storm (Mudd et al., 2020; Wang et al., 2020a). Based on this premise, data mining of the literature for ARDS studies in which measures of cytokine levels in BALF and blood are provided in the same patients yielded the results presented in **Tables 1** and **2**.

**Table 1:** Systemic and bronchoalveolar lavage fluid (BALF) cytokines in ARDS of pulmonary origin.

	**Systemic**	**BALF**	**Time**	**n**	**Study**
**MCP1**					
Pneumonia	80	**3,000**	3 hrs	5	1
COVID	1,660	**3,470**	3 days	4	2
**IL-8**					
Pneumonia	60	**300**	3 days	47	3
Pneumonia	370	**463**	1 day	44	4
Pneumonia	30	**761**	3 hrs	5	1
COVID	1,250	**3,375**	3 days	4	2
**IL-1β**					
Pneumonia	51	**95**	1 day	44	4
Pneumonia	7	**100**	3 hrs	5	1
**TNF-α**					
Pneumonia	15	**77**	1 day	44	4
Pneumonia	15	**300**	3 hrs	5	1
Pneumonia	**30**	ND*	3 days	47	3
COVID	ND	ND	3 days	4	2
**IL-6**					
Pneumonia	1,500	**3,000**	3 days	49	3
Pneumonia	220	**283**	1 day	44	4
Pneumonia	317	**919**	3 hrs	5	1
COVID	896	3,786**	21-23 days	1	5
COVID	**1,560**	1,300	3 days	4	2

Values are the means in pg/ml. The means were either given or approximated from measurements provided (transparent grid overlay). Time: time of sample collections in hours/days after start of mechanical ventilation.*ND, not detected; **IL-6 levels in pleural effusion = 18,000 pg/ml. **Study 1** (Osaki et al., 2010): ARDS, bilateral infiltrates, 1/5 patients died. **Study 2** (Ronit et al., 2021): ARDS, bilateral infiltrates, lymphopenia, 2/4 patients died. **Study 3** (Schutte et al., 1996): ARDS, microorganisms detected in BALF, 42% of patients died. When BALF levels of IL-8 and IL-6 were corrected for urea, the predicted levels in alveolar fluid were 10-fold higher, indicating local production of the two cytokines. **Study 4** (Lee et al., 2010): acute respiratory failure due to severe pneumonia, 34/44 patients died. BALF cytokine levels exceeded systemic levels regardless of the comparisons made (e.g., survivors vs non-survivors). **Study 5** (Wang et al., 2020a): ARDS, septic shock, multiple organ failure, the patient died. Based on the Il-6 gradient from lungs to blood, proposed that “the local (cytokine) storm may be worse than the systemic storm”. Compartments with higher levels in bold font.

**Table 2:** Systemic and bronchoalveolar lavage fluid (BALF) cytokines in ARDS of non-pulmonary origin.

	**Systemic**	**BALF**	**Time**	**n**	**Study**
**IL-8**					
	**3,525**	480	2 hrs	20	1
	630	**3540**	17 days	6	2
	20	**250**	3 days	10	3
**IL-1β**					
	460	**11,900**	17 days	6	2
	**364**	267	2 hrs	23	4
**TNF-α**					
	370	**3,900**	17 days	6	2
	204	**463**	2 hrs	23	4
	**40**	ND*	3 days	10	3
**IL-6**					
	388	**538**	2 hrs	20	1
	880	**9,780**	17 days	6	2
	400	**4,000**	3 days	10	3

Values are the means in pg/ml, except study 4 in which values are the medians in pg/ml. The values were either given or approximated from measurements provided (transparent grid overlay). Time: time of sample collections in hours/days after start of mechanical ventilation. *ND, detected. **Study 1** (Bouros et al., 2004): ARDS, respiratory failure, bilateral infiltrates; diagnosis: trauma (9), pneumonia (3), sepsis (2), transfusion (2), pancreatitis (2), intoxication (1), burns (1); 14/20 patients died. Regardless of comparisons made (e.g., survivors vs non-survivors) IL-6 levels in BALF exceeded systemic levels, while systemic levels of IL-8 exceeded their BALF levels. **Study 2** (Meduri et al., 1995): ARDS, respiratory failure, lung PMN infiltrates;diagnosis: pneumonia (3), aspiration (1), urosepsis (3), intra-abdominal infection; (1) 4/8 patients died. Time of sampling varied from 5 to 30 days after ARDS with a mean of 17 days. **Study 3** (Schutte et al., 1996): ARDS, respiratory failure, lung PMN infiltrates; diagnosis: sepsis (nonpulmonary origin) (7), shock (3); 6/10 patients died. **Study 4** (Agouridakis et al., 2002): ARDS, respiratory failure, bilateral infiltrates; diagnosis: trauma (9), pneumonia (5), sepsis (2), transfusion (2), pancreatitis (2), intoxication (1), burns (1); 12/23 patients died. Compartment with higher levels in bold font.

**Table 1** presents data from ARDS of pulmonary origin, while **Table 2** presents data from extrapulmonary causes. Of note, none of the studies specifically addressed a potential lung-to-systemic cytokine gradient. Further, the sample sizes are rather small, particularly for the two COVID-19 cases in **Table 1**. Finally, no attempt is made to establish statistical differences. Despite these limitations, there is a notable trend for higher levels of inflammatory cytokines in BALF than plasma of ARDS patients in which the inciting event was of pulmonary origin (**Table 1**). This lung-to-systemic gradient is not as evident in ARDS of non-pulmonary origin (**Table 2**). Analogous results are obtained in experimental models of lung inflammation and injury. When the inciting factor is a direct insult to the lungs, the BALF levels of cytokines exceed their blood levels (**Table 3**). Of note, dismantling of NETs within the airspace (intratracheal DNase), reduces LPS-induced alveolar damage. This maneuver decreases the cytokine levels in both compartments, while maintaining a BALF-to-lung cytokine gradient (**Table 3**). By contrast, when lung inflammation/injury is a result of an indirect insult (e.g., peritonitis), a BALF-to-blood cytokine gradient is not evident (**Table 4**). Collectively, a BALF-to-blood cytokine gradient is noted in ARDS and animal models when the inciting event is of lung origin. Thus, despite the limited data from COVID-19 cases, it seems likely that a lung-centric cytokine storm may characterize the ARDS of COVID-19.

**Table 3:** Systemic and bronchoalveolar lavage fluid (BALF) cytokines in animal models of direct lung injury.

**`**	**Systemic**	**BALF**	**Time**	**n**	**Study**
**IL-8**					
Acid	330	6,700**	6 hrs	10	1
**MIP-2**					
VILI	ND*	**200**	2 hrs	4	2
**IL-1β**					
VILI	ND	**30**	2 hrs	4	2
**IL-6**					
LPS	2,125	**3,050**	24 hrs	16	3
DNase/LPS	270	**1,670**	24 hrs	6	3
**TNF-α**					
VILI	ND	ND	2 hrs	4	2
LPS	450	**1,460**	24 hrs	6	3
DNase/LPS	280	**500**	24 hrs	6	3

Values are means in pg/ml. The values were either given or approximated from measurements provided (transparent grid overlay). Time: time of sample collections in hours after direct insult to lungs. *ND, not detected. **Samples from distal small bronchi, presumed to represent alveolar fluid, contained 40,500 pg/ml IL-8. **Study 1** (Folkesson et al., 1995): Hydrochloric acid given intratracheally to rabbits. Indices of lung injury: lung edema and systemic hypoxia, PMN infiltration. All rabbits died within 12 -14 hours after lung injury. **Study 2** (Ricard et al., 2001): ventilator-induced lung injury in rats (VILI; 42 ml/kg tidal volume). Index of lung injury: Increased protein levels in BALF. **Study 3** (Liu et al., 2016): lipopolysaccharide (LPS) given intratracheally to mice. Indices of lung injury: interstitial edema, PMN infiltration, hemorrhage, NET components in BALF and lung tissue. Intratracheal DNase reduced NET formation and lung injury. Compartment with higher levels in bold font.

**Table 4:** Systemic and bronchoalveolar lavage fluid (BALF) cytokines in animal models of indirect lung injury.

	**Systemic**	**BALF**	**Time**	**n**	**Study**
**MCP-1**					
DH	**600**	25	12hrs	7	1
**KC**					
DH	**1500**	100	12hrs	7	1
**IL-6**					
CLP	20,000	20,000	24 hrs	6	3
CLP	**9,000**	5,000	6 hrs	6	2
**IL-1β**					
CLP	90	**180**	24 hrs	6	3
CLP	50	**60**	6 hrs	6	2
**TNF-α**					
CLP	**280**	220	24 hrs	6	3
CLP	**50**	25	6 hrs	6	2

Values are means in pg/ml. The values were either given or approximated from measurements provided (transparent grid overlay). Time: time of sample collections in hours after insult. **Study 1** (Kalbitz et al., 2016): mice were subjected to a double-hit (DH)insult consisting of bilateral lung contusion followed 24 hrs later by cecal ligation and perforation. Indices of lung injury: protein in BALF, MPO activity in lung homogenates. **Study 2** (Wang et al., 2019): mice were subjected to cecal ligation and perforation (CLP). Indices of lung injury: inflammatory cell infiltration, alveolar damage, and edema in lung tissue. CLP also injured the heart, liver, and kidneys. 9/20 mice died by 24 hrs after CLP and 15/20 died by 7 days. **Study 3** (Wang et al., 2020b): mice were subjected to cecal ligation and perforation (CLP). Indices of lung injury: inflammatory cell infiltration, alveolar damage, and edema in lung tissue. Compartment with high levels in bold font.

Inherent in experimental models of lung inflammation/injury is a defined time interval from insult to assessment of endpoints. Chemokines (e.g., IL-8, MIP-2) are detected as early as 2 – 6 hours after direct injury to the lungs, exceeding plasma levels by at least 20-fold (**Table 3**). Further, the acid-induced lung injury (pulmonary edema and impaired oxygenation) is associated with the presence of neutrophils in the BALF (Folkesson et al., 1995). Both neutrophil recruitment and lung injury are prevented by therapeutic (post-acid) blockade of IL-8. These observations are consistent with the following scenario. Acid stimulates lung epithelium and/or macrophages to generate IL-8, which attracts neutrophils to the lungs, where they are activated and cause injury (Folkesson et al., 1995).

There are a few issues of relevance to COVID-19 that warrant attention. When BALF and plasma samples are obtained early after admission to ICU, a lung-to-blood gradient for the chemokines, IL-8 is detected (**Table 1**); correspondingly, the BALF also contains activated monocytes and neutrophils (Ronit et al., 2021). In the same samples, no such gradient was detected for the pro-inflammatory cytokines, TNF-α or IL-6 (Ronit et al., 2021). However, in a longitudinal study of one patient with protracted COVID-19 (over 3 weeks), a 4-fold BALF to blood gradient for IL-6 was attained just days before death (**Table 1**). Of note, in pleural effusion samples obtained concurrently, the level of IL-6 was 20-fold greater than in plasma (Wang et al., 2020a). Further, assuming a progression in disease severity over time, additional insight is gained by comparisons of moderate to severe cases of COVID-19. Chemokines (e.g., IL-8) dominate the BALF profile in moderate cases, whereas pro-inflammatory cytokines (e.g., TNF-α, IL-6) are prevalent in more severe cases (Liao et al., 2020). The BALF of moderate COVID-19 patients is enriched with macrophages, while BALF of severe cases is enriched with neutrophils, with the neutrophil count being directly related to the levels of IL-8 (Pandolfi et al., 2020). Thus, the SARS-CoV-2 infection of the lungs appears to follow the expected trajectory of an inflammatory response (Folkesson et al., 1995).

## Lung-Centric Immunothrombosis

The recruitment and activation of neutrophils can result in the formation of neutrophil extracellular traps, or NETs (**Figure 1**). NETs are an extruded web of decondensed chromatin DNA decorated with granule-derived proteases and antimicrobials (Papayannopoulos, 2018; Boeltz et al., 2019). The formation of GSDMD pores in neutrophil membranes facilitates the release of NETs to the extracellular space (Tall and Westerterp, 2019; Chen et al., 2020). In lung tissues of fatal COVID-19 cases, NETs have been detected in close association with damaged alveoli (Middleton et al., 2020; Radermecker et al., 2020; Veras et al., 2020). Further, complexes of NETs and platelets, as well as thrombi, have been noted in the lung microvasculature (Iba et al., 2020; Leppkes et al., 2020; Radermecker et al., 2020). Collectively, these findings are consistent with immunothrombosis, a pathway linking innate immunity with thrombosis (Gaertner and Massberg, 2016; Iba et al., 2020; Nakazawa and Ishizu, 2020; Loo et al., 2021). The homeostatic function of this pathway is to limit pathogen spread.

As a caveat, the formation of thrombi may occlude the affected microvasculature and result in ischemic injury (Ackermann et al., 2020). An IL-1β/NET/coagulation pathway has been invoked in thrombogenesis in a cohort of acute coronary syndrome patients with high circulating CRP (Liberale et al., 2019). The lungs are particularly susceptible to immunothrombosis, given the readily available pool of neutrophils (Granton et al., 2018) and platelets (Lefrançais et al., 2017). In this scenario, a viral-induced inflammation promotes the formation of NETs by activated neutrophils. The NETs serve as a scaffold for sequestering activated platelets and components of the coagulation cascade (Mcdonald et al., 2017), setting the stage for the generation of thrombi. Occlusive thrombi within the pulmonary vasculature have been noted in fatal COVID-19 cases (Ackermann et al., 2020; Leppkes et al., 2020; Middleton et al., 2020; Radermecker et al., 2020; Rapkiewicz et al., 2020). Of note, anticoagulants (e.g., heparinoids) are advocated to alleviate the hypercoagulation state of COVID-19 (Bikdeli et al., 2020; Connors and Levy, 2020; Leentjens et al., 2021). However, given the potential for bleeding, specific guidelines for thromboprophylaxis in these patients await the outcome of ongoing clinical trials (Leentjens et al., 2021).

Circulating DNases can degrade the DNA backbone of NETs and may serve as an endogenous regulatory mechanism to limit immunothrombosis (Jiménez-Alcázar et al., 2017; Mcdonald et al., 2017). In murine models of sepsis, dismantling of NETs by DNases reduces occlusive intravascular clots in the lungs and improves survival (Jiménez-Alcázar et al., 2017; Lefrançais et al., 2018). In ARDS patients (all causes), the severity of disease and lethality is related to the ratio of plasma NETs/DNases (Lefrançais et al., 2018). A similar situation appears to exist in COVID-19 patients, circulating NETs are increased with a corresponding decrease in DNase levels (Lee et al., 2021).

In fatal cases of COVID-19 microvascular thrombi and necrotic injury in organs remote from the lungs have been noted on autopsies (Ackermann et al., 2020; Rapkiewicz et al., 2020; Tang et al., 2020). Potential mechanisms include spill-over of either the virus or host cytokines from damaged lungs into the systemic circulation. However, there is little evidence that viable SARS-CoV-2 becomes blood-borne (Andersson et al., 2020; Lamouroux et al., 2020; Vivanti et al., 2020). Further, circulating levels of proinflammatory cytokines do not reach levels considered detrimental to tissues (Kox et al., 2020; Mudd et al., 2020; Sinha et al., 2020). Whether the pulmonary NET-mediated immunothrombosis of COVID-19 can impact remote organs is not clear at present. While NETs and NET-associated thrombi have consistently been found in the lungs of fatal cases, their presence in remote organs is equivocal (Leppkes et al., 2020; Radermecker et al., 2020). Alternatively, while NET remnants (presumably due to DNase-induced turnover) occurs in COVID-19 (Leppkes et al., 2020), the initiated hypercoagulative and thrombotic milieu may be a source of NET-independent remote organ involvement (Connors and Levy, 2020). Given the paucity of information on this issue, any conclusions regarding mechanisms of remote organ injury in COVID-19 are rather speculative.

## Potential Lung-Centric Therapy

The lung-centric inflammatory response prompts consideration of the potential clinical utility of bronchoscopy and BALF. BALF analyses are currently used to provide a microbiological diagnosis of COVID-19 in suspected cases, but in which nasopharyngeal swabs are negative for viral RNA (Mondoni et al., 2020; Patrucco et al., 2020). In addition, BALF analyses can also direct specific antimicrobial therapy and bronchoscopy can be used to clear the bronchial passage (Bruyneel et al., 2020). Of interest from a therapeutic perspective are the results of a murine study in which local lung inflammation and injury was induced by intratracheal LPS (**Table 3**). The recruited and activated PMN generated NETs within the airspace, as evidenced by NET markers in BALF (Liu et al., 2016). Unlike NETs formed in blood vessels, which are rapidly cleared (in part *via* DNase), NETs in the airspace are stable structures (Lefrançais et al., 2018). Exogenous intratracheal DNase reduces NETs in the BALF with a corresponding reduction in both BALF and systemic cytokines (**Table 3**). Of relevance to the ARDS of COVID-19, hypoxemic patients on ventilators were given nebulized recombinant human DNase I as part of their therapeutic regimen; no adverse effects were noted and the potential for benefit has prompted several clinical trials (Weber et al., 2020).

The possibility of directly targeting cytokines generated within the lungs of COVID-19 patients is attractive for at least two reasons. First, based on the lung-centric cytokine storm, reducing lung cytokines or their activity would also reduce corresponding circulating levels and their effects on remote organs. Second, intrapulmonary therapeutics would be less prone to off-target systemic effects. Ideally, an initial assessment of the BALF levels of cytokines in COVID-19 patients should be made as soon as possible after diagnosis. This would allow for therapeutic targeting of relevant cytokines. While early intervention would maximize patient benefit, limiting lung inflammation even in advanced cases is desirable.

The rapidly expanding repertoire of animal models of COVID-19 (Cleary et al., 2020; Muñoz-Fontela et al., 2020) should facilitate the translation of experimental findings to the clinical realm. While many of the models lack specific features noted in the human disease (e.g., lethality) (Ehaideb et al., 2020), some do have notable similarities to severe COVID-19. In this regard, transgenic mice expressing ACE2 under the cytokeratin 18 promoter (K18-hACE2) hold promise (Winkler et al., 2020; Yinda et al., 2021). These mice exhibit severe lung inflammation and dysfunction, lymphopenia, evidence of coagulopathy, remote organ involvement, and fatality. As it appears to be the case in COVID-19, viral RNA can be detected in remote organs of K18-hACE2 mice despite the absence of viremia (Yinda et al., 2021). Since murine models are readily amenable to experimental manipulation, this enigma may be resolved in future studies. Further, experiments to more directly address lung-centric immunothrombosis (e.g., NET formation, BALF to blood cytokine gradient) in these and other genetically tractable mice (Jarnagin et al., 2021) should provide a basis for lung-centric directed therapy.

## Author Contributions

Conceptualization: PK and HF. Literature search: PK, HF, and SK. Data analysis and interpretation: PK and HF. Drafting the article: PK and HF. Critical revision of the article: PK, HF, SK, AY, EA-M, and KA-K. All authors contributed to the article and approved the submitted version.

## Conflict of Interest

The authors declare that the research was conducted in the absence of any commercial or financial relationships that could be construed as a potential conflict of interest.

## References

[B1] Ackermann M. Verleden S. E. Kuehnel M. Haverich A. Welte T. Laenger F.. (2020). Pulmonary Vascular Endothelialitis, Thrombosis, and Angiogenesis in Covid-19. N Engl. J. Med. 383, 120–128. 10.1056/NEJMoa2015432 32437596 PMC7412750

[B2] Agouridakis P. Kyriakou D. Alexandrakis M. G. Prekates A. Perisinakis K. Karkavitsas N.. (2002). The Predictive Role of Serum and Bronchoalveolar Lavage Cytokines and Adhesion Molecules for Acute Respiratory Distress Syndrome Development and Outcome. Respir. Res. 3, 25. 10.1186/rr193 12537603 PMC150513

[B3] Andersson M. I. Arancibia-Carcamo C. V. Auckland K. Baillie J. K. Barnes E. Beneke T.. (2020). SARS-Cov-2 RNA Detected in Blood Products From Patients With COVID-19 Is Not Associated With Infectious Virus. Wellcome Open Res. 5, 181. 10.12688/wellcomeopenres.16002.2 33283055 PMC7689603

[B4] Barnes B. J. Adrover J. M. Baxter-Stoltzfus A. Borczuk A. Cools-Lartigue J. Crawford J. M.. (2020). Targeting Potential Drivers of COVID-19: Neutrophil Extracellular Traps. J. Exp. Med. 217, e20200652. 10.1084/jem.20200652 32302401 PMC7161085

[B5] Bikdeli B. Madhavan M. V. Jimenez D. Chuich T. Dreyfus I. Driggin E.. (2020). Covid-19 and Thrombotic or Thromboembolic Disease: Implications for Prevention, Antithrombotic Therapy, and Follow-Up: Jacc State-of-the-Art Review. J. Am. Coll. Cardiol. 75, 2950–2973. 10.1016/j.jacc.2020.04.031 32311448 PMC7164881

[B6] Blanco-Melo D. Nilsson-Payant B. E. Liu W. C. Uhl S. Hoagland D. Moller R.. (2020). Imbalanced Host Response to SARS-CoV-2 Drives Development of COVID-19. Cell 181, 1036–1045.e1039. 10.1016/j.cell.2020.04.026 32416070 PMC7227586

[B7] Boeltz S. Amini P. Anders H. J. Andrade F. Bilyy R. Chatfield S.. (2019). To NET or Not to NET:current Opinions and State of the Science Regarding the Formation of Neutrophil Extracellular Traps. Cell Death Differ. 26, 395–408. 10.1038/s41418-018-0261-x 30622307 PMC6370810

[B8] Bouros D. Alexandrakis M. G. Antoniou K. M. Agouridakis P. Pneumatikos I. Anevlavis S.. (2004). The Clinical Significance of Serum and Bronchoalveolar Lavage Inflammatory Cytokines in Patients at Risk for Acute Respiratory Distress Syndrome. BMC Pulm. Med. 4, 1–9. 10.1186/1471-2466-4-6 15315713 PMC516781

[B9] Broz P. Pelegrin P. Shao F. (2020). The Gasdermins, a Protein Family Executing Cell Death and Inflammation. Nat. Rev. Immunol. 20, 143–157. 10.1038/s41577-019-0228-2 31690840

[B10] Bruyneel M. Gabrovska M. Rummens P. Roman A. Claus M. Stevens E.. (2020). Bronchoscopy in COVID-19 Intensive Care Unit Patients. Respirology 25, 1313–1315. 10.1111/resp.13932 32844524 PMC7460941

[B11] Carsana L. Sonzogni A. Nasr A. Rossi R. S. Pellegrinelli A. Zerbi P.. (2020). Pulmonary Post-Mortem Findings in a Series of COVID-19 Cases From Northern Italy: A Two-Centre Descriptive Study. Lancet Infect. Dis. 20, 1135–1140. 10.1016/S1473-3099(20)30434-5 32526193 PMC7279758

[B12] Castelli V. Cimini A. Ferri C. (2020). Cytokine Storm in COVID-19: “When You Come Out of the Storm, You Won’t Be the Same Person Who Walked in”. Front. Immunol. 11, 2132. 10.3389/fimmu.2020.02132 32983172 PMC7492381

[B13] Chen K. W. Demarco B. Broz P. (2020). Beyond Inflammasomes: Emerging Function of Gasdermins During Apoptosis and Netosis. EMBO J. 39, e103397. 10.15252/embj.2019103397 31793683 PMC6960442

[B14] Chowdhury M. E. H. Rahman T. Khandakar A. Al-Madeed S. Zughaier S. M. Doi S. A. R.. (2021). An Early Warning Tool for Predicting Mortality Risk of COVID-19 Patients Using Machine Learning. Cognit. Comput., 1–16. 10.1007/s12559-020-09812-7 PMC8058759 33897907

[B15] Cleary S. J. Magnen M. Looney M. R. Page C. P. (2020). Update on Animal Models for COVID-19 Research. Br. J. Pharmacol. 177, 5679–5681. 10.1111/bph.15266 33140409 PMC7707085

[B16] Connors J. M. Levy J. H. (2020). COVID-19 and Its Implications for Thrombosis and Anticoagulation. Blood 135, 2033–2040. 10.1182/blood.2020006000 32339221 PMC7273827

[B17] De Biasi S. Meschiari M. Gibellini L. Bellinazzi C. Borella R. Fidanza L.. (2020). Marked T Cell Activation, Senescence, Exhaustion and Skewing Towards TH17 in Patients With COVID-19 Pneumonia. Nat. Commun. 11, 3434. 10.1038/s41467-020-17292-4 32632085 PMC7338513

[B18] Deshmukh V. Motwani R. Kumar A. Kumari C. Raza K. (2021). Histopathological Observations in COVID-19: A Systematic Review. J. Clin. Pathol. 74, 76–83. 10.1136/jclinpath-2020-206995 32817204

[B19] Diao B. Wang C. Tan Y. Chen X. Liu Y. Ning L.. (2020). Reduction and Functional Exhaustion of T Cells in Patients With Coronavirus Disease 2019 (COVID-19). Front. Immunol. 11, 827. 10.3389/fimmu.2020.00827 32425950 PMC7205903

[B20] Ehaideb S. N. Abdullah M. L. Abuyassin B. Bouchama A. (2020). Evidence of a Wide Gap Between COVID-19 in Humans and Animal Models: A Systematic Review. Crit. Care 24, 594. 10.1186/s13054-020-03304-8 33023604 PMC7537968

[B21] Fajgenbaum D. C. June C. H. (2020). Cytokine Storm. New Engl. J. Med. 383, 2255–2273. 10.1056/NEJMra2026131 33264547 PMC7727315

[B22] Ferreira A. C. Soares V. C. De Azevedo-Quintanilha I. G. Dias S. D. S. G. Fintelman-Rodrigues N. Sacramento C. Q.. (2021). Sars-CoV-2 Engages Inflammasome and Pyroptosis in Human Primary Monocytes. Cell Death Discov. 7, 43. 10.1038/s41420-021-00428-w 33649297 PMC7919254

[B23] Folkesson H. G. Matthay M. A. Hebert C. A. Broaddus V. C. (1995). Acid Aspiration-Induced Lung Injury in Rabbits is Mediated by Interleukin-8-Dependent Mechanisms. J. Clin. Invest. 96, 107–116. 10.1172/JCI118009 7615779 PMC185178

[B24] Gaertner F. Massberg S. (2016). Blood Coagulation in Immunothrombosis-At the Frontline of Intravascular Immunity. Semin. Immunol. 28, 561–569. 10.1016/j.smim.2016.10.010 27866916

[B25] Giamarellos-Bourboulis E. J. Netea M. G. Rovina N. Akinosoglou K. Antoniadou A. Antonakos N.. (2020). Complex Immune Dysregulation in COVID-19 Patients With Severe Respiratory Failure. Cell Host Microbe. 27, 992–1000 e1003. 10.1016/j.chom.2020.04.009 32320677 PMC7172841

[B26] Granton E. Kim J. H. Podstawka J. Yipp B. G. (2018). The Lung Microvasculature Is a Functional Immune Niche. Trends Immunol. 39, 890–899. 10.1016/j.it.2018.09.002 30253910

[B27] Hadjadj J. Yatim N. Barnabei L. Corneau A. Boussier J. Smith N.. (2020). Impaired Type I Interferon Activity and Inflammatory Responses in Severe COVID-19 Patients. Science 369, 718–724. 10.1126/science.abc6027 32661059 PMC7402632

[B28] Hariharan A. Hakeem A. R. Radhakrishnan S. Reddy M. S. Rela M. (2021). The Role and Therapeutic Potential of NF-kappa-B Pathway in Severe COVID-19 Patients. Inflammopharmacology 29, 91–100. 10.1007/s10787-020-00773-9 33159646 PMC7648206

[B29] Hoffmann M. Kleine-Weber H. Schroeder S. Krüger N. Herrler T. Erichsen S.. (2020). Sars-CoV-2 Cell Entry Depends on ACE2 and TMPRSS2 and Is Blocked by a Clinically Proven Protease Inhibitor. Cell 181, 271–280. e278. 10.1016/j.cell.2020.02.052 32142651 PMC7102627

[B30] Iba T. Levy J. H. Levi M. Connors J. M. Thachil J. (2020). Coagulopathy of Coronavirus Disease 2019. Crit. Care Med. 48, 1358–1364. 10.1097/CCM.0000000000004458 32467443 PMC7255402

[B31] Jarnagin K. Alvarez O. Shresta S. Webb D. R. (2021). Animal Models for SARS-Cov2/Covid19 Research-a Commentary. Biochem. Pharmacol. 188, 114543. 10.1016/j.bcp.2021.114543 33812856 PMC8016548

[B32] Jiménez-Alcázar M. Rangaswamy C. Panda R. Bitterling J. Simsek Y. J. Long A. T.. (2017). Host DNases Prevent Vascular Occlusion by Neutrophil Extracellular Traps. Science 358, 1202–1206. 10.1126/science.aam8897 29191910

[B33] Kalbitz M. Karbach M. Braumueller S. Kellermann P. Gebhard F. Huber-Lang M.. (2016). Role of Complement C5 in Experimental Blunt Chest Trauma-Induced Septic Acute Lung Injury (Ali). PloS One 11, e0159417. 10.1371/journal.pone.0159417 27437704 PMC4954719

[B34] Kelley N. Jeltema D. Duan Y. He Y. (2019). The NLRP3 Inflammasome: An Overview of Mechanisms of Activation and Regulation. Int. J. Mol. Sci. 20, 3328. 10.3390/ijms20133328 PMC6651423 31284572

[B35] Kox M. Waalders N. J. B. Kooistra E. J. Gerretsen J. Pickkers P. (2020). Cytokine Levels in Critically Ill Patients With COVID-19 and Other Conditions. Jama 324, 1565–1567. 10.1001/jama.2020.17052 PMC7489366 32880615

[B36] Lamouroux A. Attie-Bitach T. Martinovic J. Leruez-Ville M. Ville Y. (2020). Evidence for and Against Vertical Transmission for Severe Acute Respiratory Syndrome Coronavirus 2. Am. J. Obstet. Gynecol. 223, e1–91. 10.1016/j.ajog.2020.04.039 PMC7196550 32376317

[B37] Lara P. C. Macias-Verde D. Burgos-Burgos J. (2020). Age-Induced NLRP3 Inflammasome Over-activation Increases Lethality of SARS-CoV-2 Pneumonia in Elderly Patients. Aging Dis. 11, 756–762. 10.14336/AD.2020.0601 32765942 PMC7390513

[B38] Lax S. F. Skok K. Zechner P. Kessler H. H. Kaufmann N. Koelblinger C.. (2020). Pulmonary Arterial Thrombosis in COVID-19 With Fatal Outcome: Results From a Prospective, Single-Center, Clinicopathologic Case Series. Ann. Intern. Med. 173, 350–361. 10.7326/M20-2566 32422076 PMC7249507

[B39] Lee S. Channappanavar R. Kanneganti T. D. (2020). Coronaviruses: Innate Immunity, Inflammasome Activation, Inflammatory Cell Death, and Cytokines. Trends Immunol. 41, 1083–1099. 10.1016/j.it.2020.10.005 33153908 PMC7561287

[B40] Lee Y. L. Chen W. Chen L. Y. Chen C. H. Lin Y. C. Liang S. J.. (2010). Systemic and Bronchoalveolar Cytokines as Predictors of in-Hospital Mortality in Severe Community-Acquired Pneumonia. J. Crit. Care 25, 176. e177–176. e113. 10.1016/j.jcrc.2009.05.002 19592208

[B41] Leentjens J. van Haaps T. F. Wessels P. F. Schutgens R. E. Middeldorp S. (2021). COVID-19-Associated Coagulopathy and Antithrombotic Agents—Lessons After 1 Year. Lancet Haematol. S2352-3026 (21), 00105–8. 10.1016/S2352-3026(21)00105-8 PMC8078884 33930350

[B42] Lee Y. Y. Park H. H. Park W. Kim H. Jang J. G. Hong K. S.. (2021). Long-Acting Nanoparticulate DNase-1 for Effective Suppression of SARS-CoV-2-Mediated Neutrophil Activities and Cytokine Storm. Biomaterials 267, 120389. 10.1016/j.biomaterials.2020.120389 33130319 PMC7583619

[B43] Lefrançais E. Mallavia B. Zhuo H. Calfee C. S. Looney M. R. (2018). Maladaptive Role of Neutrophil Extracellular Traps in Pathogen-Induced Lung Injury. JCI Insight 3, e98178. 10.1172/jci.insight.98178 PMC5821185 29415887

[B44] Lefrançais E. Ortiz-Muñoz G. Caudrillier A. Mallavia B. Liu F. Sayah D. M.. (2017). The Lung Is a Site of Platelet Biogenesis and a Reservoir for Haematopoietic Progenitors. Nature 544, 105–109. 10.1038/nature21706 28329764 PMC5663284

[B45] Leppkes M. Knopf J. Naschberger E. Lindemann A. Singh J. Herrmann I.. (2020). Vascular Occlusion by Neutrophil Extracellular Traps in COVID-19. EBioMedicine 58, 102925. 10.1016/j.ebiom.2020.102925 32745993 PMC7397705

[B46] Liao M. Liu Y. Yuan J. Wen Y. Xu G. Zhao J.. (2020). Single-Cell Landscape of Bronchoalveolar Immune Cells in Patients With COVID-19. Nat. Med. 26, 842–844. 10.1038/s41591-020-0901-9 32398875

[B47] Liberale L. Holy E. W. Akhmedov A. Bonetti N. R. Nietlispach F. Matter C. M.. (2019). Interleukin-1β Mediates Arterial Thrombus Formation *via* NET-Associated Tissue Factor. J. Clin. Med. 8, 2072. 10.3390/jcm8122072 PMC6947515 31779200

[B48] Liu Q. Shi Y. Cai J. Duan Y. Wang R. Zhang H.. (2020). Pathological Changes in the Lungs and Lymphatic Organs of 12 COVID-19 Autopsy Cases. Natl. Sci. Rev. 7, 1868–1878. 10.1093/nsr/nwaa247 PMC7543449 34676085

[B49] Liu S. Su X. Pan P. Zhang L. Hu Y. Tan H.. (2016). Neutrophil Extracellular Traps Are Indirectly Triggered by Lipopolysaccharide and Contribute to Acute Lung Injury. Sci. Rep. 6, 37252. 10.1038/srep37252 27849031 PMC5110961

[B50] Loo J. Spittle D. A. Newnham M. (2021). Covid-19, Immunothrombosis and Venous Thromboembolism: Biological Mechanisms. Thorax. horaxjnl-2020-216243. 10.1136/thoraxjnl-2020-216243 33408195

[B51] Lucas C. Wong P. Klein J. Castro T. B. Silva J. Sundaram M.. (2020). Longitudinal Analyses Reveal Immunological Misfiring in Severe COVID-19. Nature 584, 463–469. 10.1038/s41586-020-2588-y 32717743 PMC7477538

[B52] Manjili R. H. Zarei M. Habibi M. Manjili M. H. (2020). COVID-19 as an Acute Inflammatory Disease. J. Immunol. 205, 12–19. 10.4049/jimmunol.2000413 32423917 PMC7333792

[B53] Mcdonald B. Davis R. P. Kim S. J. Tse M. Esmon C. T. Kolaczkowska E.. (2017). Platelets and Neutrophil Extracellular Traps Collaborate to Promote Intravascular Coagulation During Sepsis in Mice. Blood 129, 1357–1367. 10.1182/blood-2016-09-741298 28073784 PMC5345735

[B54] Meduri G. U. Headley S. Tolley E. Shelby M. Stentz F. Postlethwaite A. (1995). Plasma and BAL Cytokine Response to Corticosteroid Rescue Treatment in Late ARDS. Chest 108, 1315–1325. 10.1378/chest.108.5.1315 7587435

[B55] Mehta P. Mcauley D. F. Brown M. Sanchez E. Tattersall R. S. Manson J. J.. (2020). COVID-19: Consider Cytokine Storm Syndromes and Immunosuppression. Lancet 395, 1033–1034. 10.1016/S0140-6736(20)30628-0 32192578 PMC7270045

[B56] Merad M. Martin J. C. (2020). Pathological Inflammation in Patients With COVID-19: A Key Role for Monocytes and Macrophages. Nat. Rev. Immunol. 20 (6), 355–362. 10.1038/s41577-020-0331-4 32376901 PMC7201395

[B57] Middleton E. A. He X. Y. Denorme F. Campbell R. A. Ng D. Salvatore S. P.. (2020). Neutrophil Extracellular Traps Contribute to Immunothrombosis in COVID-19 Acute Respiratory Distress Syndrome. Blood 136, 1169–1179. 10.1182/blood.2020007008 32597954 PMC7472714

[B58] Milewska A. Kula-Pacurar A. Wadas J. Suder A. Szczepanski A. Dabrowska A.. (2020). Replication of Severe Acute Respiratory Syndrome Coronavirus 2 in Human Respiratory Epithelium. J. Virol. 94, e00957-20. 10.1128/JVI.00957-20 32434888 PMC7375387

[B59] Mondoni M. Sferrazza Papa G. F. Rinaldo R. Faverio P. Marruchella A. D’arcangelo F.. (2020). Utility and Safety of Bronchoscopy During the SARS-CoV-2 Outbreak in Italy: A Retrospective, Multicentre Study. Eur. Respir. J. 56, 2002767. 10.1183/13993003.02767-2020 32859682 PMC7453732

[B60] Morris G. Bortolasci C. C. Puri B. K. Olive L. Marx W. O’neil A.. (2021). Preventing the Development of Severe COVID-19 by Modifying Immunothrombosis. Life Sci. 264, 118617. 10.1016/j.lfs.2020.118617 33096114 PMC7574725

[B61] Mudd P. A. Crawford J. C. Turner J. S. Souquette A. Reynolds D. Bender D.. (2020). Distinct Inflammatory Profiles Distinguish COVID-19 From Influenza With Limited Contributions From Cytokine Storm. Sci. Adv. 6, eabe3024. 10.1126/sciadv.abe3024 33187979 PMC7725462

[B62] Muñoz-Fontela C. Dowling W. E. Funnell S. G. P. Gsell P. S. Riveros-Balta A. X. Albrecht R. A.. (2020). Animal Models for COVID-19. Nature 586, 509–515. 10.1038/s41586-020-2787-6 32967005 PMC8136862

[B63] Nakazawa D. Ishizu A. (2020). Immunothrombosis in Severe COVID-19. EBioMedicine 59, 102942. 10.1016/j.ebiom.2020.102942 32810824 PMC7428773

[B64] Osaki Y. Maehara Y. Sato M. Hoshino A. Yamamoto K. Nagao T.. (2010). Analysis of Cytokine/Chemokine Levels in Bronchoalveolar Lavage Fluids From Patients With Acute Respiratory Distress Syndrome. J. Jpn. Soc. Intensive Care Med. 17, 179–184. 10.3918/jsicm.17.179

[B65] Pandolfi L. Fossali T. Frangipane V. Bozzini S. Morosini M. D’amato M.. (2020). Broncho-Alveolar Inflammation in COVID-19 Patients: A Correlation With Clinical Outcome. BMC Pulm. Med. 20, 301. 10.1186/s12890-020-01343-z 33198751 PMC7668012

[B66] Papayannopoulos V. (2018). Neutrophil Extracellular Traps in Immunity and Disease. Nat. Rev. Immunol. 18, 134–147. 10.1038/nri.2017.105 28990587

[B67] Patrucco F. Albera C. Bellocchia M. Foci V. Gavelli F. Castello L. M.. (2020). SARS-Cov-2 Detection on Bronchoalveolar Lavage: An Italian Multicenter Experience. Respiration 99, 970–978. 10.1159/000511964 33075793 PMC7649696

[B68] Phua J. Weng L. Ling L. Egi M. Lim C. M. Divatia J. V.. (2020). Intensive Care Management of Coronavirus Disease 2019 (COVID-19): Challenges and Recommendations. Lancet Respir. Med. 8, 506–517. 10.1016/S2213-2600(20)30161-2 32272080 PMC7198848

[B69] Radermecker C. Detrembleur N. Guiot J. Cavalier E. Henket M. D’emal C.. (2020). Neutrophil Extracellular Traps Infiltrate the Lung Airway, Interstitial, and Vascular Compartments in Severe COVID-19. J. Exp. Med. 217, e20201012. 10.1084/jem.20201012 32926097 PMC7488867

[B70] Rapkiewicz A. V. Mai X. Carsons S. E. Pittaluga S. Kleiner D. E. Berger J. S.. (2020). Megakaryocytes and Platelet-Fibrin Thrombi Characterize Multi-Organ Thrombosis at Autopsy in COVID-19: A Case Series. EClinicalMedicine 24, 100434. 10.1016/j.eclinm.2020.100434 32766543 PMC7316051

[B71] Ricard J. D. Dreyfuss D. Saumon G. (2001). Production of Inflammatory Cytokines in Ventilator-Induced Lung Injury: A Reappraisal. Am. J. Respir. Crit. Care Med. 163, 1176–1180. 10.1164/ajrccm.163.5.2006053 11316656

[B72] Rodrigues T. S. De Sa K. S. G. Ishimoto A. Y. Becerra A. Oliveira S. Almeida L.. (2021). Inflammasomes Are Activated in Response to SARS-CoV-2 Infection and Are Associated With COVID-19 Severity in Patients. J. Exp. Med. 218, e20201707. 10.1084/jem.20201707 33231615 PMC7684031

[B73] Ronit A. Berg R. M. G. Bay J. T. Haugaard A. K. Ahlstrom M. G. Burgdorf K. S.. (2021). Compartmental Immunophenotyping in COVID-19 Ards: A Case Series. J. Allergy Clin. Immunol. 147, 81–91. 10.1016/j.jaci.2020.09.009 32979342 PMC7581505

[B74] Schaefer I. M. Padera R. F. Solomon I. H. Kanjilal S. Hammer M. M. Hornick J. L.. (2020). In Situ Detection of SARS-CoV-2 in Lungs and Airways of Patients With COVID-19. Mod. Pathol. 33, 2104–2114. 10.1038/s41379-020-0595-z 32561849 PMC7304376

[B75] Schutte H. Lohmeyer J. Rosseau S. Ziegler S. Siebert C. Kielisch H.. (1996). Bronchoalveolar and Systemic Cytokine Profiles in Patients With ARDS, Severe Pneumonia and Cardiogenic Pulmonary Oedema. Eur. Respir. J. 9, 1858–1867. 10.1183/09031936.96.09091858 8880103

[B76] Sinha P. Matthay M. A. Calfee C. S. (2020). Is a “Cytokine Storm” Relevant to COVID-19? JAMA Internal Med. 180, 1152–1154. 10.1001/jamainternmed.2020.3313 32602883

[B77] Swanson K. V. Deng M. Ting J. P. (2019). The NLRP3 Inflammasome: Molecular Activation and Regulation to Therapeutics. Nat. Rev. Immunol. 19, 477–489. 10.1038/s41577-019-0165-0 31036962 PMC7807242

[B78] Tall A. R. Westerterp M. (2019). Inflammasomes, Neutrophil Extracellular Traps, and Cholesterol. J. Lipid Res. 60, 721–727. 10.1194/jlr.S091280 30782961 PMC6446695

[B79] Tang D. Comish P. Kang R. (2020). The Hallmarks of COVID-19 Disease. PloS Pathog. 16, e1008536. 10.1371/journal.ppat.1008536 32442210 PMC7244094

[B80] Tay M. Z. Poh C. M. Renia L. Macary P. A. Ng L. F. P. (2020). The Trinity of COVID-19: Immunity, Inflammation and Intervention. Nat. Rev. Immunol. 20, 363–374. 10.1038/s41577-020-0311-8 32346093 PMC7187672

[B81] Toldo S. Bussani R. Nuzzi V. Bonaventura A. Mauro A. G. Cannata A.. (2021). Inflammasome Formation in the Lungs of Patients With Fatal COVID-19. Inflammation Res. 70, 7–10. 10.1007/s00011-020-01413-2 PMC7572246 33079210

[B82] Veras F. P. Pontelli M. C. Silva C. M. Toller-Kawahisa J. E. De Lima M. Nascimento D. C.. (2020). Sars-CoV-2-Triggered Neutrophil Extracellular Traps Mediate COVID-19 Pathology. J. Exp. Med. 217, e20201129. 10.1084/jem.20201129 32926098 PMC7488868

[B83] Vivanti A. J. Vauloup-Fellous C. Prevot S. Zupan V. Suffee C. Do Cao J.. (2020). Transplacental Transmission of SARS-CoV-2 Infection. Nat. Commun. 11, 3572. 10.1038/s41467-020-17436-6 32665677 PMC7360599

[B84] Wang Y. M. Ji R. Chen W. W. Huang S. W. Zheng Y. J. Yang Z. T.. (2019). Paclitaxel Alleviated Sepsis-Induced Acute Lung Injury by Activating MUC1 and Suppressing TLR-4/NF-κb Pathway. Drug Des. Devel. Ther. 13, 3391–3404. 10.2147/DDDT.S222296 PMC6766586 31576113

[B85] Wang C. Kang K. Gao Y. Ye M. Lan X. Li X.. (2020a). Cytokine Levels in the Body Fluids of a Patient With COVID-19 and Acute Respiratory Distress Syndrome: A Case Report. Ann. Intern. Med. 173, 499–501. 10.7326/L20-0354 32422085 PMC7224607

[B86] Wang Y. M. Qi X. Gong F. C. Chen Y. Yang Z. T. Mao E. Q.. (2020b). Protective and Predictive Role of Mucin1 in Sepsis-Induced ALI/ARDS. Int. Immunopharmacol. 83, 106438. 10.1016/j.intimp.2020.106438 32247267

[B87] Weber A. G. Chau A. S. Egeblad M. Barnes B. J. Janowitz T. (2020). Nebulized In-Line Endotracheal Dornase Alfa and Albuterol Administered to Mechanically Ventilated COVID-19 Patients: A Case Series. Mol. Med. 26, 91. 10.1186/s10020-020-00215-w 32993479 PMC7522910

[B88] Winkler E. S. Bailey A. L. Kafai N. M. Nair S. Mccune B. T. Yu J.. (2020). Sars-CoV-2 Infection of Human ACE2-Transgenic Mice Causes Severe Lung Inflammation and Impaired Function. Nat. Immunol. 21, 1327–1335. 10.1038/s41590-020-0778-2 32839612 PMC7578095

[B89] Yang D. Chu H. Hou Y. Chai Y. Shuai H. Lee A. C.-Y.. (2020). Attenuated Interferon and Proinflammatory Response in SARS-CoV-2–Infected Human Dendritic Cells Is Associated With Viral Antagonism of STAT1 Phosphorylation. J. Infect. Dis. 222, 734–745. 10.1093/infdis/jiaa356 32563187 PMC7337793

[B90] Yinda C. K. Port J. R. Bushmaker T. Offei Owusu I. Purushotham J. N. Avanzato V. A.. (2021). K18-hACE2 Mice Develop Respiratory Disease Resembling Severe COVID-19. PloS Pathog. 17, e1009195. 10.1371/journal.ppat.1009195 33465158 PMC7875348

[B91] Zhao Q. Meng M. Kumar R. Wu Y. Huang J. Deng Y.. (2020). Lymphopenia Is Associated With Severe Coronavirus Disease 2019 (COVID-19) Infections: A Systemic Review and Meta-Analysis. Int. J. Infect. Dis. 96, 131–135. 10.1016/j.ijid.2020.04.086 32376308 PMC7196544

[B92] Zhao C. Zhao W. (2020). Nlrp3 Inflammasome—A Key Player in Antiviral Responses. Front. Immunol. 11, 211. 10.3389/fimmu.2020.00211 32133002 PMC7040071

[B93] Zheng H.-Y. Zhang M. Yang C.-X. Zhang N. Wang X.-C. Yang X.-P.. (2020). Elevated Exhaustion Levels and Reduced Functional Diversity of T Cells in Peripheral Blood May Predict Severe Progression in COVID-19 Patients. Cell. Mol. Immunol. 17, 541–543. 10.1038/s41423-020-0401-3 32203186 PMC7091621

[B94] Zhu N. Wang W. Liu Z. Liang C. Wang W. Ye F.. (2020). Morphogenesis and Cytopathic Effect of SARS-CoV-2 Infection in Human Airway Epithelial Cells. Nat. Commun. 11, 3910. 10.1038/s41467-020-17796-z 32764693 PMC7413383

